# Does detection range matter for inferring social networks in a benthic shark using acoustic telemetry?

**DOI:** 10.1098/rsos.170485

**Published:** 2017-09-06

**Authors:** Johann Mourier, Nathan Charles Bass, Tristan L. Guttridge, Joanna Day, Culum Brown

**Affiliations:** 1 Department of Biological Sciences, Macquarie University, North Ryde, New South Wales 2109, Australia; 2 Laboratoire d'excellence ‘CORAIL’, PSL Research University, EPHE-UPVD-CNRS, USR 3278 CRIOBE, 66860 Perpignan, France; 3 Bimini Biological Field Station Foundation, 15 Elizabeth Drive, South Bimini, Bahamas; 4 Taronga Conservation Society Australia, Mosman, New South Wales 2088, Australia

**Keywords:** acoustic telemetry, social networks, fish, shark, detection range

## Abstract

Accurately estimating contacts between animals can be critical in ecological studies such as examining social structure, predator–prey interactions or transmission of information and disease. While biotelemetry has been used successfully for such studies in terrestrial systems, it is still under development in the aquatic environment. Acoustic telemetry represents an attractive tool to investigate spatio-temporal behaviour of marine fish and has recently been suggested for monitoring underwater animal interactions. To evaluate the effectiveness of acoustic telemetry in recording interindividual contacts, we compared co-occurrence matrices deduced from three types of acoustic receivers varying in detection range in a benthic shark species. Our results demonstrate that (i) associations produced by acoustic receivers with a large detection range (i.e. Vemco VR2W) were significantly different from those produced by receivers with smaller ranges (i.e. Sonotronics miniSUR receivers and proximity loggers) and (ii) the position of individuals within their network, or centrality, also differed. These findings suggest that acoustic receivers with a large detection range may not be the best option to represent true social networks in the case of a benthic marine animal. While acoustic receivers are increasingly used by marine ecologists, we recommend users first evaluate the influence of detection range to depict accurate individual interactions before using these receivers for social or predator–prey studies. We also advocate for combining multiple receiver types depending on the ecological question being asked and the development of multi-sensor tags or testing of new automated proximity loggers, such as the Encounternet system, to improve the precision and accuracy of social and predator–prey interaction studies.

## 1. Introduction

Determining animal encounters or contact rates can be central in ecological studies, for example in determining social structure, mating behaviour, predator–prey interactions and information or disease transmission [1–4]. Contact between animals in a social context can be represented as a social network. The social network analysis (SNA) framework is based on recording and integrating observations of multiple pairs of individuals which are analysed using a set of powerful statistical metrics at the individual, group or population level [5]. SNA has proved valuable to address and quantify multiple biological processes in animal populations ranging from preferred associations and patterns of assortment [6], the role of personality [7] and indirect connections in social structure [8], the processes of transmission of parasites [3] or culture [9], network resilience and robustness [10] or the role of social [11] or genetic [12] inheritance of social network properties.

While technology to measure contact between animals has significantly improved in terrestrial studies [1,13], it has received little attention in aquatic ecosystems (but see [14,15]). Indeed, directly observing wild animals, particularly in an aquatic environment, is challenging when individuals are hard to identify or when focal subjects are too elusive to get accurate repeated observations. Moreover, current technologies, such as Global Positioning System (GPS), used to track individuals and construct social networks in terrestrial animal populations are not applicable in aquatic systems. Consequently, most animal social network studies in the marine environment have relied on repeated direct observations of interactions between identified individuals [6,16–18].

While the number of SNAs has considerably increased in terrestrial animals and marine mammals [19], it remains in its infancy in marine fish [17]. This is primarily due to difficulties in following and identifying aquatic animals underwater for long periods and defining appropriate sampling periods for animals that are hard to see. However, recent studies have investigated the sociality of bentho-pelagic shark species (juvenile *Negaprion brevirostris* [16], adult *Carcharhinus melanopterus* [6,10] and *Scyliorhinus canicula* [20]). Such studies require animals to be uniquely identified. Telemetry is a potential method in which the spatio-temporal position of a tagged animal can be remotely and continuously recorded within networks of fixed ultrasonic acoustic receivers [21], thereby allowing indirect estimates of their encounters or interactions with conspecifics, predators or prey. Subsequently, networks of receivers have been recently proposed as a method to map the co-occurrences and social interactions in free-ranging marine fishes [14,21–24] and build social networks. The combination of automatic telemetry with network analysis, therefore, presents great potential for investigating the behaviour of species that are difficult to observe directly [23,24]. Although acoustic telemetry has been used successfully to monitor shark behaviour, such an integrated approach has had limited application (but see Guttridge *et al*. [15] for proximity loggers; Stehfest *et al*. [22] and Jacoby *et al*. [23] for Vemco VR2W acoustic receivers; and Armansin *et al*. [25] for Vemco Positioning System (VPS)).

Arrays of terrestrial RFID receivers to track electronically tagged birds are ideal to document their social network inferred from the arrival and departure of individuals from the small detection range of feeders [26]. By contrast, VR2W acoustic receivers often have large and variable detection ranges (up to 800 m radius around the device [27,28]), which can result in sharks being detected simultaneously from up to 1600 m apart. Using multiple receivers can provide information into synchronicity in movements of individuals and in turn on some form of social structure in mobile animals [23]. However, the large scale and variability of detection range in VR2W receivers brings in the questions as to whether they can be used to accurately report individual gregariousness and interactions as previously suggested [24]. For example, when can two individuals detected together be considered associating? At what detection range do co-occurrences become meaningful? There can be multiple scales of social grouping, and the choice of the spatial resolution will be dependent on the question at hand. As research questions should drive edge definition in social networks [29,30], it is important to consider whether different scale-dependent methods of network construction are appropriate to estimate realistic social encounters, especially in large marine benthic animals, which spend most of the day resting on the bottom. Animal social networks are often constructed from proximity data on the spatio-temporal co-occurrence of identifiable subjects as a proxy for interaction networks [5,31], but the validity of this assumption has rarely been tested despite the importance of defining edges in social networks [29,30]. Despite the appealing use of acoustic receivers to automatically build social networks in aquatic animals, such method might not be effective because of broad detection ranges. It is therefore critical to understand how comparable networks are when constructed using these methods and how or if the network properties are affected according to variations in detection ranges.

In this study, we aimed to confront the resolution issue of spatio-temporal data collection from individual associations using three different types of acoustic underwater receivers with varying detection ranges. We took advantage of an ongoing study of the seasonal mating aggregation of a benthic shark (the Port Jackson shark *Heterodontus portusjacksoni*) in eastern Australia to compare social networks built from detections at different types of ultrasonic receivers that were deployed in Jervis Bay (New South Wales, Australia). The Port Jackson shark is a good model as this species forms large mating aggregations on near shore rocky reefs for which they show very high levels of philopatry and seasonal residency [32]. We used three types of receivers: (i) Vemco VR2W acoustic receivers are the most commonly used and record the presence of animals fitted with acoustic transmitters within a radius varying between 50 and 800 m depending on environmental conditions, (ii) Sonotronics miniSUR are also ultrasonic receivers, but they have the capability to adjust their detection range (set to 10 and 60 m in this study) by modifying the gain, and (iii) proximity loggers are animal-borne receivers and record other tagged animals in close vicinity of the focal individual (less than 10 m). In the context of a breeding aggregation of a benthic shark, we questioned whether all types of receivers of different detection ranges returned similar edge weight and individual positions in social networks despite capturing associations at different spatial scales.

## 2. Material and methods

### 2.1. Study site and design

**Figure 1.**
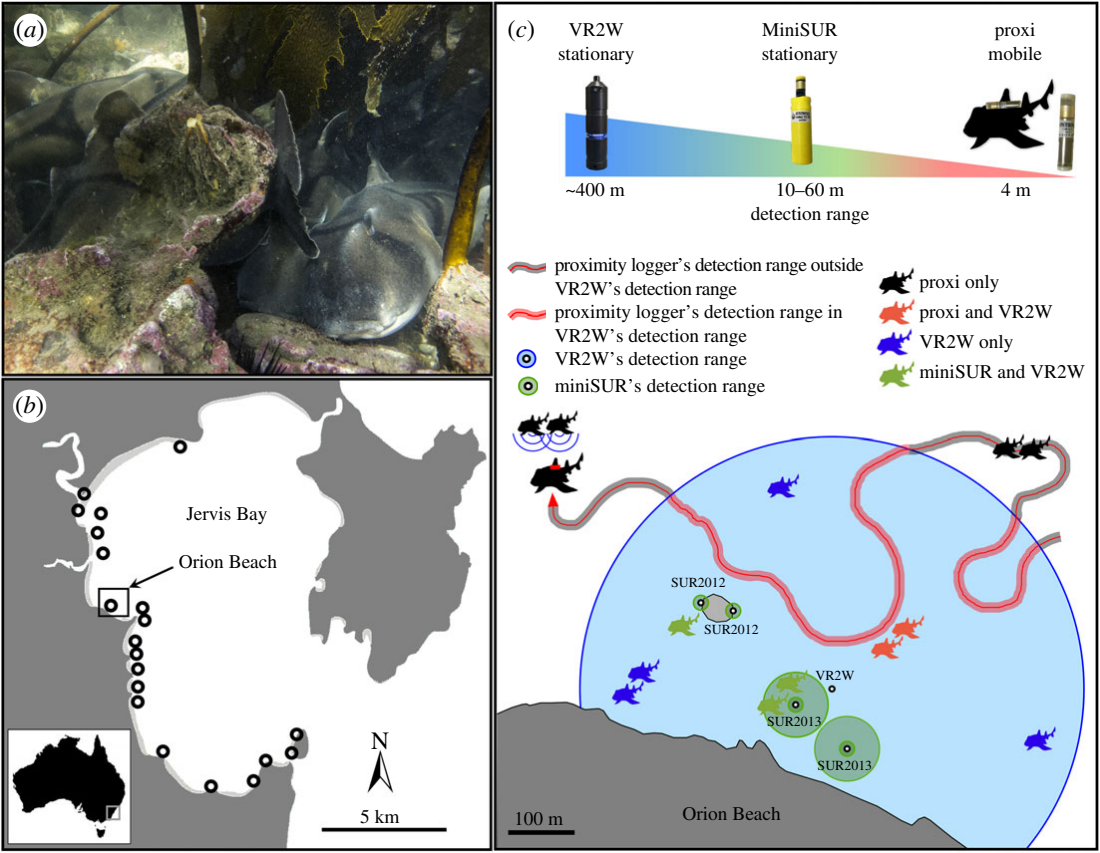
Conceptual framework of the study design. (*a*) Group of individual Port Jackson sharks resting together at their mating aggregation. (*b*) Locations of Vemco VR2W acoustic receivers deployed in Jervis Bay (NSW, Australia); each circle representing a receiver. (*c*) Zoom in Orion Beach with the location of acoustic receivers, including one VR2W acoustic receiver deployed in 2012–2013, two miniSUR receivers deployed in 2012 and two miniSUR receivers deployed in 2013. Each receiver is represented by a small white circle and larger one represents its detection range (blue for VR2W and green for miniSURs). The red path represents a fictional movement track of a shark equipped with a proximity logger able to record the presence of encountered tagged sharks both inside (red path area) and outside (grey path area) the VR2W's detection range. Colours of shark silhouettes represent the different recording possibilities by the receivers: black sharks for individuals or groups recorded by the proximity logger only outside of the VR2W's detection range, red sharks are detected by the proximity logger, green sharks are recorded by the miniSURs and blue sharks are recorded by the VR2W receiver only. Blue and red sharks are also recorded by the VR2W receiver. Note also that sharks recorded by the VR2W receiver only (blue sharks) can be either within a group or solitary due to the large detection range.

The study was conducted in Jervis Bay in New South Wales, Australia. This bay hosts large seasonal mating aggregations of Port Jackson sharks, a benthic species endemic to Australia, in which both males and females undertake yearly migrations from their foraging grounds in southern Australia to return to the same reef in Jervis Bay to breed [32]. Proximity loggers (ARX-RX1, Sonotronics, Inc., Tucson, AZ, USA) were attached to seven individual sharks in 2012, whereas Vemco VR2W (Vemco, Halifax, Nova Scotia, Canada) and Sonotronics miniSUR (Sonotronics, Inc.) acoustic receivers were deployed in 2012 and 2013 (one VR2W and two miniSUR at the main study site Orion Beach each year; figure 1).

### 2.2. Tagging procedures

Adult Port Jackson sharks were hand-captured by SCUBA divers and snorkelers, and slowly brought to the surface. They were then transported to shore in a canoe, where they were immediately transferred to a trough containing fresh seawater. The total length (TL) of individuals was measured to the nearest centimetre. Females ranged from 91 to 129 cm TL, whereas males ranged from 88 to 113 cm TL. Individuals were sexed based on the presence or absence of claspers, fin clipped for genetic and isotopic analysis, and tagged with passive integrated transponder tags (FDXB transponders; Microchips Australia) for individual identification. Individuals were then sedated in a solution of tricaine methanesulfonate (MS-222; 150 mg ml^−1^), and an acoustic transmitter (Vemco V16, 69 kHz) was implanted in their peritoneal cavity through a 2.5 cm incision, which was then sutured using five interrupted sutures and superglue. Transmitters were programmed with a nominal delay of 90 s and an expected battery life of 2805 days. All individuals were revived and released at their site of capture. More details are provided in Bass *et al*. [32]. In this study, the subjects were eight females and 10 males tagged in 2012, and 13 females and eight males tagged in 2013.

### 2.3. VR2W acoustic receivers

Vemco VR2W acoustic receivers are the most frequently used tool in marine animal tracking [27]. These receivers are used to record the presence or movements of fishes or other aquatic fauna. They are fixed to the substrate or attached to mooring lines in mid-water and record the presence of any acoustic transmitter within a detection range that varies considerably according to environmental conditions [27,28]. The detectability radius around the receiver can vary from 50 to 800 m depending on habitat and context. In this study, VR2W receivers had a detection range of about 400 m radius [32], and we were interested in estimating the association patterns of a benthic shark during mating aggregations; Port Jackson sharks often form tight groups of several individuals which can even sit on top of each other (figure 1*a*).

### 2.4. MiniSUR receivers

Omni-directional submersible ultrasonic receivers (miniSUR, Sonotronics, Inc.) are fixed acoustic receivers that record the identity of tagged individuals as per VR2Ws, but the detection range can be selected by modifying the gain setting of the receiver, allowing co-occurrences to be defined at a finer resolution with the goal to record fish being within one to five body lengths which is regularly used to define group formation or an association [15,25]. In this study, the detection range of the miniSURs was set to 18 dB in 2012 corresponding to approximately 10 m radius according to the manufacturer (Sonotronics, Inc.). This detection range corresponds to about 10 Port Jackson shark body lengths (approx. 1–1.5 m TLs), which would pick up any shark resting in close proximity (i.e. grouping; figure 1*a*). In 2013, the gain of the miniSUR was set to shift every 5 min between 18 and 36 dB (between approx. 10 and 60 m). Range tests were conducted in the field to verify the detection range by deploying a miniSUR receiver in a fixed position and then gradually moving a Vemco V16 acoustic transmitter away from the receiver in order to determine the drop-out of detection strength. These tests confirmed an average range of 10 or 60 m depending on the settings implemented in 2012 and 2013. Note that miniSUR receivers can record and decode Vemco tags after sending the raw data to the manufacturer.

### 2.5. Sonotronics proximity loggers

Proximity loggers (Sonotronics, Inc.) are miniature omni-directional receivers that can be attached to an animal's body. In this study, the Port Jackson sharks used were 88–129 cm in TL, weighing 6–18 kg in air. The proximity tag weighed 31 g in air (16 g in seawater); this is less than 1% of the body weight of these sharks, a percentage that has been shown in other studies not to affect shark behaviour [15]. Tags were mounted to the first dorsal fin of the shark using monofilament. They are designed to log the date and time of ultrasonic transmitters set to 69 kHz frequency (Vemco V16 acoustic transmitters) moving within the receiver's detection range [15]. The gain settings can be modified to restrict detection range. For this study, the receiver sensitivity setting was arranged to detect transmitters within approximately 4 m (10–30 db), equivalent to approximately two to three Port Jackson shark body lengths. The detection range was then verified in the field following the same method described for miniSUR receivers and confirmed that it was approximately 4 m. The proximity logger data were used to collect association data for all individuals that co-occurred within its range at the same interval (figure 1). Because the individual upon which the proximity logger was mounted would be included in all association events detected, it was excluded from the network. Note that Sonotronics proximity loggers can record and decode Vemco tags after adjustment during decoding process.

### 2.6. Study design and data analysis

Our analysis is based on testing the similarity/dissimilarity of social networks built from receivers of varying detection ranges (i.e. from 4 to 400 m) and measuring association patterns at different spatial scales. Such comparative analysis was conducted according to a variety of restrictions in order to consider different spatial scales from the three receiver types. First, because the proximity receiver had the shortest monitoring period, we restricted data from miniSURs and the VR2W receiver from Orion Beach to the same monitoring period for comparison. Moreover, both of the miniSURs were deployed within the range of one VR2W receiver at Orion Beach and had a smaller detection range than the VR2W receiver (figure 1). Therefore, we would expect individuals recorded by miniSURs to also be recorded by the VR2W. The mobile proximity logger records individuals in close proximity to the focal shark, but does not provide any spatial information for these interactions. Therefore, recorded associations cannot be geolocalized, which limits the ability to appropriately compare the output with other receivers if most interactions occurred outside the VR2W's detection range. To deal with this issue, we extracted the groups inferred from the proximity logger in which individual members were also detected by the VR2W based on timestamps of groups (figure 1). Furthermore, as a final comparison, we considered the network constructed from associations at all VR2W receivers in Jervis Bay (figure 1*a*). As both males and females show high site fidelity to their home reefs during the mating season [32], this species is a good choice for testing these methods. As a result, we were able to compare five datasets in 2012: (i) co-occurrences recorded by all VR2W receivers deployed in Jervis Bay, (ii) co-occurrences recorded only by the receiver from Orion Beach where miniSUR was deployed, (iii) co-occurrences recorded by the miniSUR receivers at a 10 m detection range setting, (iv) associations recorded by the proximity loggers within the VR2W's range, and (v) total data from the proximity loggers including associations occurring outside the VR2W's range; and four datasets in 2013: (i) co-occurrences recorded by all VR2W receivers from Jervis Bay, (ii) co-occurrences recorded from the VR2W receiver from Orion Beach, (iii) co-occurrences recorded by the miniSUR receivers at a 10 m detection range setting, and (iv) co-occurrences recorded by the miniSUR receivers at a 60 m detection range setting.

Social association matrices were constructed based on the ‘gambit of the group’ approach [5,31] where groups are defined as co-occurrences of individuals within a receiver's detection range and within an arbitrary pre-defined 10-min bin (e.g. 0.00–0.09, 0.10–0.19, etc.). This also allows time for all receivers to detect animals on a minimum of six occasions based on V16 delay time (i.e. 90 s). Association strength for each dyad was calculated using the simple ratio index, where associations are scaled between 0 (never observed in the same group) and 1 (always occurred in the same group) [33]. Using the simple ratio index, the edge weight was calculated using the following equation [5]:

$$E_{\mathrm{AB}}=\frac{x}{x+y_{\mathrm{AB}}+y_{A}+y_{B}},$$

where (*x*) is the number of sampling periods where *A* and *B* co-occurred, (*y*_AB_) is the number of times both *A* and *B* were observed in the same sample but not together, *y*_A_ is the number of samples where only individual *A* was seen and *y*_B_ is the number of samples where only *B* was seen. We used an hourly sampling period to construct our networks. Network construction and analysis were performed with the *asnipe* package in R [34].

To compare the similarity of resulting association indices between methods, we first compared association matrices using a Mantel test for each receiver type. We also investigated the similarity of network structural properties by determining the consistency of weighted degree (the sum of associations from one individual) between networks produced by the different receivers. We used methods described in Wilson *et al*. [35] by comparing the sum of the variances for individuals' network position (weighted degree) across observed networks (SV_O_) with the sum of individual variances in positions from randomized networks (SV_R_). Individuals were ranked within each network according to their weighted degree and scaled between 0 and 1. Individual positions were thus relative to all others in the network, with small values of SV_O_ indicating a similar relative ranking across repeated samples. Significance was assessed by comparing the SV_O_ of weighted degree for each network comparison with a frequency distribution of SV_R_ values generated from 10 000 randomizations of the observed data. Randomization procedures were conducted following a data-stream randomization procedure in asnipe R package [34] using the default algorithm using one swap and 10 000 permutations. This procedure is usually used to determine the consistency of individuals with repeated sample networks [26]. The randomization procedure was also used to test for randomness of associations for each network by comparing observed mean and coefficient of variation (CV) of association indices with those of the 10 000 random networks [5].

## 3. Results

### 3.1. Data comparison

Of the seven proximity loggers deployed, only three were retrieved, from which only one provided reliable data; the other two loggers either did not accurately code timestamp data or presented early battery failure and therefore could not be used in the analysis. In 2012, data for all receivers were restricted to the period during which the retained proximity logger provided data: from 25 August to 10 October 2012. In 2013, data from miniSUR and VR2W were recorded from 23 August to 21 October 2013, and data from miniSURs were filtered for gain 18 and 64 dB (corresponding to 10 and 60 m detection ranges, respectively). For all receiver comparisons, we restricted our analyses to individuals present in each network: 15 individuals in 2012 and 18 in 2013.

### 3.2. Association indices

Networks all presented mean observed association indices significantly larger than those of their respective random networks (*p* < 0.001). They also all showed observed CV significantly higher than random ones (at least *p* < 0.01), except for the proximity logger restricted to the VR2W in Orion Beach in 2012 (*p* = 0.491). These results suggest that all receivers (except the VR2W) were able to demonstrate that Port Jackson sharks showed some form of non-random associations measured by co-occurrence.

In 2012, Mantel tests showed that association indices from the proximity logger and miniSUR receivers, with a detection range of 4 and 10 m, respectively, were significantly correlated (*r* = 0.780, *p* = 0.001; table 1). Association data produced by the proximity logger restricted to the VR2W's range and in its totality were significantly correlated (*r* = 0.889, *p* = 0.001; table 1). Similarly, association matrices produced by all VR2W receivers in Jervis Bay produced were correlated to the one produced when only considering the VR2W from Orion Beach (*r* = 0.869, *p* = 0.001; table 1). However, comparisons between VR2Ws and either proximity (inside or outside VR2W range) or miniSUR receivers in 2012 revealed that association matrices were not significantly correlated in both cases (table 1).

**Table 1.** Statistical output from comparison of Port Jackson shark network properties between receivers.

		association matrices similarity		degree consistency		
year	receiver comparison	Mantel *r*	*p-*value	SV_O_	SV_R_ (±CI)	*p-*value
2012	VR2W (Orion)–VR2W (all)	0.869	0.001	0.331	0.241 (0.231; 0.262)	1.000
2012	VR2W (all)–Proxi (all)	−0.163	0.827	1.689	0.976 (0.953; 1.000)	1.000
2012	VR2W (all)–Proxi (Orion)	−0.105	0.721	1.591	1.174 (1.146; 1.202)	1.000
2012	VR2W (all)–SUR-10 m	−0.167	0.827	1.643	0.434 (0.421; 0.447)	1.000
2012	VR2W (Orion)–SUR-10 m	−0.157	0.807	1.576	0.518 (0.505; 0.532)	1.000
2012	VR2W (Orion)–Proxi (VR2W)	−0.161	0.828	1.607	1.495 (1.463; 1.527)	0.972
2012	SUR-10 m–Proxi (VR2W)	0.712	0.001	0.199	0.766 (0.741; 0.790)	0.000
2012	Proxi–Proxi (VR2W)	0.889	0.001	0.158	0.919 (0.894; 0.943)	0.000
2012	Proxi–SUR-10 m	0.780	0.001	0.193	0.499 (0.488; 0.501)	0.000
2012	Proxi–VR2W (Orion)	−0.253	0.930	1.887	0.897 (0.488; 0.509)	1.000
2013	VR2W (Orion)–VR2W (all)	0.794	0.001	0.363	0.804 (0.782; 0.816)	0.000
2013	VR2W (all)–SUR-60 m	0.086	0.202	2.148	1.241 (1.225; 1.241)	1.000
2013	VR2W (all)–SUR-10 m	0.034	0.351	2.229	0.804 (0.782; 0.826)	1.000
2013	VR2W (Orion)–SUR-60 m	0.034	0.364	1.917	1.241 (1.225; 1.257)	1.000
2013	VR2W (Orion)–SUR-10 m	0.018	0.402	1.912	0.804 (0.782; 0.826)	1.000
2013	SUR-10 m–SUR-60 m	0.965	0.001	0.023	0.808 (0.782; 0.836)	0.000

In 2013, there was a significant correlation between association matrices from miniSUR of 10 and 60 m range (*r* = 0.965, *p* = 0.001; table 1). Restricting associations to Orion Beach did not significantly affect the correlation between association matrices when using VR2W receivers (*r* = 0.794, *p* = 0.001; table 1). However, association matrices built from miniSURs were not significantly correlated from those inferred from VR2W receivers in both cases (table 1).

**Figure 2.**
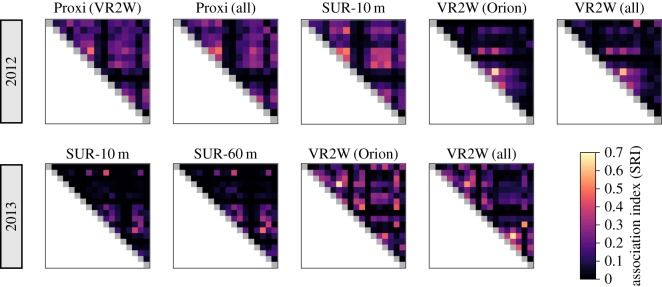
Comparative heatmaps of association matrices produced by each receiver type. For each case, same individuals (rows and columns) are ordered in a same way for 2012 and 2013 networks, respectively. Each cell of a heatmap represents a dyad. As matrices represent undirected networks, only top triangles are presented.

These observed changes in correlations were associated with a difference in the structure of the association matrices of the VR2W compared to the other receivers as shown by heatmaps of values in matrices (figure 2). In 2012, association matrices built from VR2Ws appear to underestimate association scores relative to those of miniSURs and proximity loggers (as shown by cells in figure 2 which are overall darker for VR2Ws). This may be due to the position of miniSURs in 2012, which were installed right on areas of subgroups of tagged sharks while within a wider detection range. In this case, the VR2W receiver may record both solitary and associated individuals as being part of a group, thereby weakening the relative strength of association. On the contrary in 2013, association matrices built from VR2Ws seem to overestimate association scores relative to those of miniSURs (as shown by cells in figure 2 which are overall lighter for VR2Ws). In this case, miniSURs might be placed outside the range of subgroups of tagged sharks, therefore underestimating the strength of associations because of relatively fewer detections compared with VR2W receivers.

### 3.3. Consistency in centrality rank

**Figure 3.**
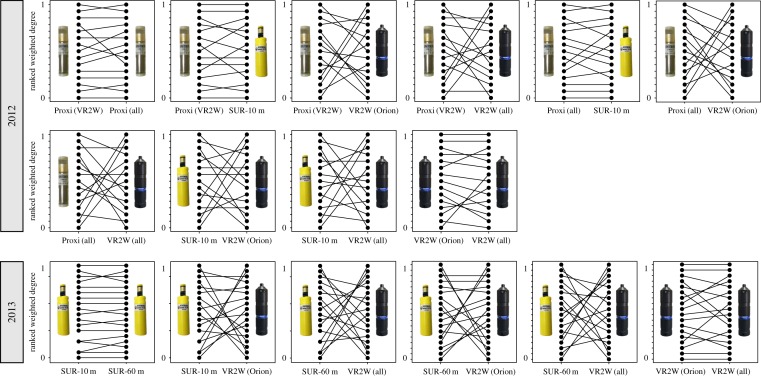
Consistency of social network data collection between receivers. Scores are scaled between 0 and 1 for all individuals and represent ranks in weighted degree of individuals in the social networks.

Observed sum of variances (SV_O_) of individual weighted degree between proximity logger and miniSUR receiver (10 m range) was statistically smaller than the randomized sum of variances (SV_R_), indicating repeatability in individual social position across methods (restricting proximity receiver to Orion Beach: SV_O_ = 0.199, *p* < 0.001 or not SV_O_ = 0.193, *p* < 0.001; table 1). Similarly, consistency was also found between miniSUR of 10 and 60 m detection range in 2013, suggesting that the same information is captured at any range within 60 m (SV_O_ = 0.023, *p* < 0.001; table 1). Consistency in rank was also found when comparing restricted data of proximity receivers (SV_O_ = 0.158, *p* < 0.001; table 1). For VR2Ws, restricting data to the VR2W at Orion Beach did not change the rank of individual in their network in 2013 (SV_O_ = 0.363, *p* < 0.001; table 1), but did in 2012 (SV_O_ = 0.331, *p* = 1.000; table 1). However, SV_O_ values were much higher, and consequently, the consistency of individuals’ ranks was not significant when comparing the network produced by the proximity logger and the miniSURs with the one constructed from the VR2W receiver (table 1). Higher and lower ranks were better conserved throughout the miniSUR and proximity receivers than the relative medium ranks (figure 3).

## 4. Discussion

Acoustic telemetry is now commonly used by marine biologists to infer movement patterns of fishes. With the development of social network tools for understanding both the spatial and social network structure in ecology, acoustic telemetry has been recently proposed as a means to investigate the social structure of aggregations by recording co-occurrences of tagged individuals at acoustic receivers [21,23,24]. Proximity networks are often used as a proxy for interaction networks, but there is a need to carefully consider the definition of edges in interaction networks and the influence of spatial scale on social interactions [30]. While acoustic telemetry has been successfully employed to infer patterns of social structuring in a mobile shark species [23], our results demonstrate that this may be species-dependent as co-occurrences inferred from receivers with a large detection range may not accurately represent social interactions at smaller spatial scales, increasing the number of false positive interactions, especially in less mobile benthic species such as Port Jackson sharks.

Our study compared three types of acoustic receivers with different detection ranges extending from 4 to about 400 m. Our results not only showed that association indices built from VR2W acoustic receivers were not correlated to those inferred from miniSUR and proximity receivers (figure 2 and table 1), but also that the position of individuals within their network (i.e. their rank in centrality) was statistically different (figure 3 and table 1). However, association indices were correlated between networks built from miniSUR and proximity receivers, and individual centrality ranks were relatively consistent. This means that, in the case of benthic animals such as Port Jackson sharks, individual associations seem relevant when recorded within a 4–60 m detection range, with a higher resolution at 4–10 m. This is not surprising as Port Jackson sharks are often found sharing refuges or gutters, forming small aggregations within a few metres (figure 1*a*) [36]. This is also in accordance with observational studies of social structure in fish and sharks where associations are generally defined as two individuals present within one to four body lengths [15,17,25]. Although our study was characterized by a low sample size, node level metrics in partial networks should predict an animal's real social position [37], especially as our study focuses on the relative difference of edge weight and centrality between network construction methods of a similar set of individuals.

Previous studies have proposed the use of passive acoustic telemetry to infer interactions in large fishes in the wild [14,22]. However, these studies used relatively large detection ranges. For instance, Holland *et al*. [14] proposed the use of acoustic ‘Business Card’ tags, which behave in a similar way to proximity loggers [15] in that they are attached to the shark and record every transmitter-attached shark swimming in proximity of the Business Card-tagged individual. This device is now commercialized under the name Vemco Mobile Transceiver (VMT) [2]. Nevertheless, detection range of the Business Card tags used by Holland *et al*. [14] was greater than 150 m and about 200 m for VMT [2]. A recent study used a similar approach to study the inter- and intra-specific encounter networks of sand tiger sharks, *Carcharias taurus*, by analysing data from VMT tags recovered from two sharks [38]. The authors defined ‘social interactions’ as two or more individuals spending two or more consecutive hours together within a circle of greater than 1000 m radius. While they argued for evidence of fission–fusion behaviour, their analysis did not test for randomness of encounters or structure of the data, a crucial step in SNA [5]. Therefore, in this case, associations may represent random encounters during seasonal migrations. In an attempt to understand the schooling patterns of the fishes, Stehfest *et al*. [22] used an array of Vemco VR2 acoustic receivers installed on artificial fish aggregating devices (FADs) to construct a network of co-occurrence of tagged yellowfin tuna (*Thunnus albacares*) visiting these FADs. The detection range of the receivers used in Stehfest *et al*. [22] was less than 600 m. While it is difficult to determine if co-occurrences within a circle of greater than 1000 m diameter are representative of biologically meaningful social interactions, the study was focused on describing the aggregative behaviour of tunas around FADs rather than real social interactions, as tunas form large schools implying that fish could be part of the same school but not necessarily associating with a particular individual. These examples reinforce the necessity to consider that research questions should drive edge definition in social networks [29,30] and that researchers need to carefully question the appropriate scale at which they need to construct their proximity networks and the analyses required to answer their specific questions.

A recent study presented a promising approach to explore social network structure from telemetry data of spatio-temporal co-occurrences [23]. While associations are also defined within the detection range of VR2W acoustic receivers, the method proposed is based on the fact that mobile animals such as sharks can form strong associations if they move between receivers together (and are continually detected on the same receivers and in different clustering events), which can be demonstrated by extracting the timing and directionality of dyadic interactions from the data [23,24]. Mobility and sociality of the focal species may, however, influence the likelihood of depicting real associations via such large range receivers and the number of receivers used. For instance, assessment of interactions in benthic species with low mobility, such as Port Jackson sharks which often rest in dense groups stacked one on top of the other (figure 1*a*), likely requires a relatively lower number of receivers and a smaller detection range than species such as reef sharks that follow and form mobile social groups over larger spatial scales. Studying co-occurrences can provide interesting and important contributions for our understanding of population dynamics [23,39], but caution is required when interpreting them as real social interactions. New technologies such as Encounternet, which is an automated telemetry system combining animal radio tags and wireless stations, can improve the creation of social networks [40,41], although this technology has rarely been tested underwater [42]. Another approach is to use the Vemco Radio-Acoustic Positioning (VRAP) system or the more recent VPS to record the spatial position of individual sharks and subsequently infer associations between individuals [25,43]. This is certainly a more accurate resolution that is similar to the use of GPS trackers in terrestrial studies and is expected to produce more realistic interactions, but remains relatively complex and costly to implement. Inferring social interactions from spatial positioning may also require further development in testing randomness of positioning [44] to remove potential unwanted by-products of spatial overlap, especially in benthic sharks that spend long periods resting on the bottom.

In our study, VR2W receivers were not able to capture co-occurrences at an appropriate spatial scale to infer social associations from Port Jackson sharks, which reside in the fact that it likely not only records real associations but also isolated solitary individuals inside the receiver range, both of which would be assigned to a group as illustrated in figure 1. In the latter case, assigning lone individuals to groups is clearly an error. The degree of mobility likely influences the rate of false positives as low mobility will increase detection probability and as a result increase assigned associations. This is especially the case for benthic species such as Port Jackson sharks [32] or wobbegong sharks [25] that spend most of their time sitting on the bottom, reducing the likelihood of encountering other individuals away from their resting area, and where social interactions may occur within one or two body lengths (i.e. approx. 3 m). In this study, we used V16 acoustic transmitters which have the strongest acoustic signal of the tags from this manufacturer, enabling a VR2W acoustic receiver to detect the V16 at a distance of about 400 m. One solution to reduce this distance could use lower-powered tags (e.g. V9 or V13) to produce similar detection ranges to the other systems tested in our study. Future work employing simulations re-creating data using different detection methods for several species of different mobility should help in better determining the influence of detection range for inferring real social associations.

Our study also demonstrates that using different types of receivers can contribute to investigating different aspects of the behavioural ecology of the Port Jackson shark. While using an optimal VPS receiver design was not possible due to budget limitations, we used a large network of VR2W acoustic receivers to document large-scale movement patterns of sharks within Jervis Bay and along the east Australian coastline [32], while deploying miniSUR acoustic receivers with a smaller detection range at specific mating aggregation sites to document patterns of associations between individuals of this benthic shark (figure 1). The lack of differences between networks constructed from all VR2Ws and restricted to the VR2W from Orion Beach may be because Port Jackson sharks only transited through other receivers before settling at Orion Beach (i.e. 99% of detections occurred at Orion Beach); therefore, most interactions occurred at Orion Beach. This would be a typical pattern of any species with high site fidelity. Our design could have been improved by reducing the power of the tags used in order to reduce the detection range of VR2W acoustic receivers to make it similar to the miniSURs. However, such reduction would have impacted the effectiveness of our large-scale migration study [32]. Our preliminary assessment of the benefit of using receivers with a small detection range to study the social network of our study benthic shark species suggests that increasing the number of miniSUR receivers at our aggregation site in future studies will likely improve the precision of our inference of the social network.

Proximity loggers [15] remain the most robust technique for inferring meaningful social interactions; however, they still suffer from the need to recover the receiver from the animal to download the data, which is challenging even in low mobile species such as demonstrated by our study, although some solutions to release and retrieve devices are under development [45]. Moreover, they do not provide any spatial information on recorded interactions, although they can be combined with other tags that do, and return a network of encounters centred around one individual. However, complemented with the VR2W receiver array as implemented in our study, spatial information can be recorded for some associations. The Business Card tags have the convenience of being able to be geolocalized within a network of acoustic receivers, but also need to be recovered to retrieve the data [14]. The optimal system for inferring social networks of marine organisms would be a combination of the two technologies, with mobile receivers recording the tagged individuals encountered within a reduced range and transferring the data to fixed listening stations in proximity [14], although this would represent a technological challenge due to time taken to download the data. Alternatively, proximity receivers could communicate with another animal-borne device providing location of the animal and remotely transferring the data from the proximity logger. This idea is under development with VMT communicating with Service Argos via Bluetooth to remotely transmit data [46]. Developing a similar technology with short-range proximity receivers will greatly improve our ability to record intra- and inter-specific interactions in the marine environment. Recently, the proximity logger technology used to infer social networks of birds [40,41] has been adapted to the aquatic context [42]. Briefly, it consists of three device types: (i) a set of small tags emitting individually coded high frequency radio signals, receives signals from other tags and its proximity, and logs perceived encounters in an on-board memory, (ii) a set of wireless base stations, fixed at known positions and recording encounters with tags, uploading the logs stored in the tags' memory and transmitting information between tags and the third component of the system, and (iii) an interface between the user and the system (i.e. a transmitter/receiver node mounted on a laptop) collects the data from the base stations. Such promising method requires testing in marine environments as it may be challenging in some conditions, but provides a promising tool to improve fine-scale association patterns in fishes.

## 5. Conclusion

Our study provides an example of the limitations and precautions that marine ecologists need to take into account when attempting to depict the social structure of fish aggregations in the marine environment using acoustic telemetry. The ecology of the species should be considered to define the scale of biologically meaningful interactions between individuals, especially its mobility as marine organisms can socialize in different ways [47]. These issues are also applicable to terrestrial studies as similar problems are found for certain highly mobile or rarely detected species. Our study provides evidence that using a network of VR2W acoustic receivers may not be judicious in exploring the social network of a benthic shark species at its mating aggregation due to potential low rate of movements over large spatial scales. We therefore encourage marine ecologists to think about the ecological research questions considered and the scales and contexts at which they can be answered [29]. Broad networks of acoustic receivers with large detection ranges (e.g. VR2Ws) can be used to construct co-occurrence networks to investigate population dynamics, particularly for mobile species that do not stay in one place for long periods of time [23], while reduction of tag power can contribute to get closer to realistic contact by reducing a detection range. Alternatively, they can be used in a triangulation set-up to achieve high spatial resolution positioning (e.g. VPS), although this set-up is more complicated and costly. Likewise, networks of receivers with a smaller detection range (e.g. miniSURs) are likely useful to build more realistic interaction networks and investigate other specific social mechanisms such as courtship, social learning or diffusion of information or disease. Combining these receiver types can represent an optimal design, using, for example, VR2Ws to monitor large-scale population dynamics and individual movement patterns and deploying miniSUR at specific restricted aggregation sites to monitor association patterns. Proximity loggers are likely the most accurate method to infer small-scale interactions in marine animals, but technological drawbacks still weaken their effectiveness. The use of multi-sensor tagging is likely a good way forward [48] in providing complementary information. Promising technological developments such as Encounternet's adaptation to the marine environment [42] or the development of new generations of proximity loggers that facilitate the retrieving of data via new communicating systems will not only benefit social interaction studies in marine environments, but also the study of key ecological processes such as predator–prey interactions.

## Data accessibility

Data are available at: http://dx.doi.org/10.5061/dryad.20864 [49].
